# The Association between Endometriomas and Ovarian Cancer: Preventive Effect of Inhibiting Ovulation and Menstruation during Reproductive Life

**DOI:** 10.1155/2015/751571

**Published:** 2015-08-30

**Authors:** Giovanni Grandi, Angela Toss, Laura Cortesi, Laura Botticelli, Annibale Volpe, Angelo Cagnacci

**Affiliations:** ^1^Department of Obstetrics Gynecology and Pediatrics, Obstetrics and Gynecology Unit, Azienda Ospedaliero Universitaria Policlinico of Modena, Via del Pozzo 71, 41124 Modena, Italy; ^2^Department of Oncology, Haematology and Respiratory Disease, Azienda Ospedaliero Universitaria Policlinico of Modena, Via del Pozzo 71, 41124 Modena, Italy; ^3^Department of Laboratory Medicine and Pathology, Azienda Ospedaliero Universitaria Policlinico of Modena, Via del Pozzo 71, 41124 Modena, Italy

## Abstract

Although endometriosis frequently involves multiple sites in the pelvis, malignancies associated with this disease are mostly confined to the ovaries, evolving from an endometrioma. Endometriomas present a 2-3-fold increased risk of transformation in clear-cell, endometrioid, and possibly low-grade serous ovarian cancers, but not in mucinous ovarian cancers. These last cancers are, in some aspects, different from the other epithelial ovarian cancers, as they do not appear to be decreased by the inhibition of ovulation and menstruation. The step by step process of transformation from typical endometrioma, through atypical endometrioma, finally to ovarian cancer seems mainly related to oxidative stress, inflammation, hyperestrogenism, and specific molecular alterations. Particularly, activation of oncogenic KRAS and PI3K pathways and inactivation of tumor suppressor genes PTEN and ARID1A are suggested as major pathogenic mechanisms for endometriosis associated clear-cell and endometrioid ovarian cancer. Both the risk for endometriomas and their associated ovarian cancers seems to be highly and similarly decreased by the inhibition of ovulation and retrograde menstruation, suggesting a common pathogenetic mechanism and common possible preventive strategies during reproductive life.

## 1. Introduction

Endometriosis is a common estrogen-dependent disease of reproductive age that affects up to 15% of women [1]. This figure can increase in particular setting, like infertile subjects or women requiring hormonal contraceptives [2]. Endometriosis is linked to pelvic pain, though it is sometimes completely asymptomatic, and infertility. Pelvic pain can be represented by dysmenorrhea, dyspareunia, chronic pelvic pain, dyschezia, and dysuria, and it can affect patients quality of life [3]. The burden of endometriosis during reproductive life changes after the menopause, when ovarian steroid hormones finish stimulating lesions and the major issue is due to the risk of malignant transformation [4].

Endometriosis is characterized by the presence and/or the growth of endometrial tissue (both glands and stroma) outside the uterine cavity that causes a chronic inflammation, inside or outside the pelvis [1]. In spite of about 90 years of research, pathogenesis of this disease is still largely unknown. The most widely accepted theory for endometriosis development in general remains the one initially proposed by Sampson in 1927 [5]. Sampson suggested that the disease originates from endometrial cells regurgitated through the Fallopian tubes during menstruation. This theory is supported by some anatomical common findings: endometriosis is more prevalent in patients with Müllerian congenital anomalies [6], in uteri with a retrograde pattern of myometrial contractility [7], or in particular uterine conformations [8]. On the other hand, this theory does not fit with all the cases of endometriosis, like those in subjects with Rokitansky-Kuster-Hauser syndrome that are without a functional endometrium, suggesting that it is unlikely that tubal regurgitation is the only mechanism implicated in the endometriosis development [9]. It is likely that endometriosis is not a single disease but it is composed by different entities with completely different pathogeneses. Indeed, three different entities of endometriosis were traditionally described: ovarian, peritoneal, and rectovaginal endometriosis [10]. Although endometriosis frequently involves multiple sites in the pelvis, malignancies associated with this disease are mostly confined to the ovaries, evolving from an endometrioma [11]. Reasons prompting the malignancy to develop only form ovarian endometriosis are largely unknown but probably due to the particular microenvironment, present at this specific site.

The aim of this review is to highlight the interesting relation between ovarian endometriomas and endometriosis-associated ovarian cancer (EAOC): starting from their pathogenesis, evaluating in parallel their relationships with menstruation and ovulation, and ending with proposals for possible common preventive strategies.

## 2. Ovarian Endometrioma

### 2.1. Pathogenesis of Ovarian Endometrioma

Ovarian endometriomas, also known as “chocolate cysts,” are benign ovarian cyst containing thick, old blood that appears as a brown fluid. The pathogenesis of ovarian endometrioma is a continuous source of controversy. The theories proposed by Hughesdon [12], Brosens et al. [13], Nezhat et al. [14], and Vercellini et al. [15] are in agreement about the origin of the cystic content: the regurgitated endometrium. Conversely, the mechanism of cyst development is different among these theories.

Hughesdon in 1957 [12] suggested that endometrial implants, located on the surface of the ovary, cause a gradual invagination of the ovarian cortex, which results in a pseudocyst. Brosens et al. [13], in agreement with Hughesdon, reported menstrual shedding and blood accumulation at the site of the implants through ovariscopy. On the other hand, Nezhat et al. [14] proposed that endometriomas may develop as a result of secondary involvement of functional ovarian cysts, while Vercellini et al. [15] hypothesized the development specifically from hemorrhagic corpora lutea, firstly suggesting a possible relation between ovulation and endometriomas.

Nisolle and Donnez in 1997 published a completely different hypothesis about the development of the endometrioma: a celomic metaplasia of invaginated superficial ovarian epithelium in typical glandular epithelium and stroma, thus excluding the possible involvement of retrograde menstruation in endometrioma's pathogenesis [16].

**Figure 1:**
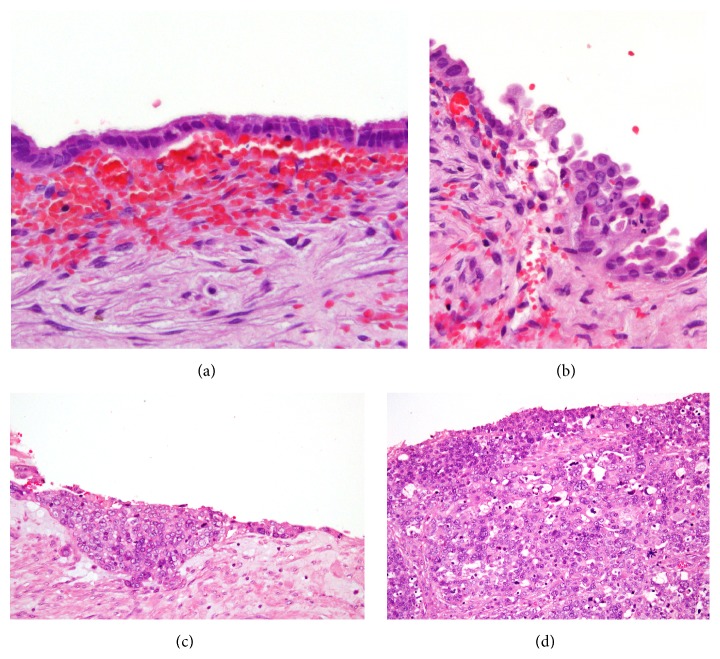
Specimens from our institution. (a) Typical endometrioma (20x EE). (b) Endometrioma with moderate atypia: initial hyperplasia and cellular atypias (40x EE). (c) Endometrioma with severe atypia: marked hyperplasia and more evident cellular atypias (20x EE). (d) Endometrioid carcinoma (40x EE).

**Figure 2:**
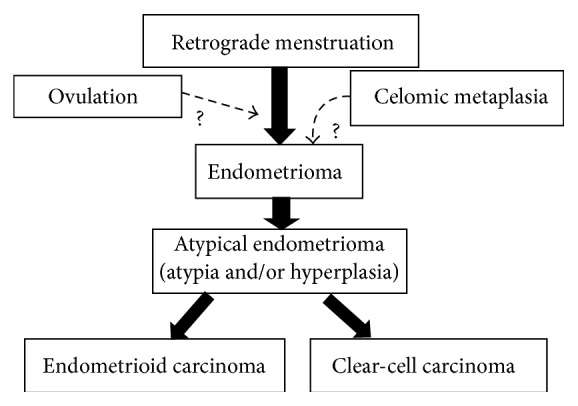
The proposed step by step process of transformation from retrograde menstruation to typical endometrioma, through atypical endometrioma, and finally to endometrioid or clear-cell ovarian cancer.

From the histopathologic point of view, “atypical endometriomas” are regarded as the precursor lesions for most endometrioid and clear-cell ovarian cancers. The risk of malignant transformation of atypical endometriomas is about 4-fold increase. There are histological evidences of transition from endometriosis, through atypical endometriosis, to EAOC [17]. The most important features in the endometrial epithelium for the study of malignant transformation are cytologic atypia and/or hyperplasia [18]. Moderate atypia (often reactive) is characterized by a layer of flattened or cuboidal cells with a large, pleomorphic, and hyperchromatic nucleus; vice versa severe atypia, which can be considered a premalignant lesion, is characterized by cells with a pale or pleomorphic and hyperchromatic nucleus, eosinophilic cytoplasm, and intraluminal projections (Figure 1). Thomas and Campbell [19] classified atypical endometriosis on the basis of the following histologic criteria: large nucleus, hyperchromatic or pale, with accentuated pleomorphism, decrease in the cytoplasm/nucleus relation, and cellular stratification. The presence of hyperplasia in the glandular epithelium is less common but is described in some atypical endometriomas (Figure 1). In a review of a large series of studies, approximately 8% of endometriomas are reported to contain atypical endometriosis [20]. Increased awareness of the characteristics of atypical endometriomas will improve early detection of patients with endometriosis who are at risk of EAOC. The proposed step by step process of transformation from retrograde menstruation to ovarian cancer is presented in Figure 2.

### 2.2. Effect of Ovulation Inhibition on Endometrioma Development

Endometriosis is strongly correlated with a history of infertility and nulliparity [1]. Unfortunately, measures for the prevention of endometrioma before the clinical diagnosis are not known.

It was initially assumed that past oral contraceptives users are at higher risk for endometriosis. The explanation of this paradigm is that dysmenorrhea, as a reason to initiate estroprogestins, is significantly more common in women with endometriosis than in women without the disease (“the chicken or the egg causality dilemma”) [21].

A good in vivo model of “zero-time” for the study of the endometrioma development is the period after a conservative surgery for stripping of an endometrioma and the risk of recurrence. Several studies have demonstrated the important reduction of cyst recurrence after surgery for cystectomy in case of prolonged ovulation inhibition, like during the use of estroprogestin contraceptive pills. A recent meta-analysis found a recurrent endometrioma one year after surgery in 8% of “always” oral contraceptive users and in 34% of women who do not use it (pooled odds ratio 0.12; 95% confidence interval 0.05–0.29) [22]. This rate of recurrence, also during constant ovulation inhibition, may indicate that ovulation is not the only mechanism involved in endometrioma development. However, the presence of bias such as the presence of residual cyst after a noncomplete surgery cannot be excluded.

A progestin (dienogest) administered at dosages capable of inhibiting ovulation demonstrated being efficient in reducing the stage of endometriosis, evaluated laparoscopically by the revised American Fertility Society (rAFS) score [23]. In addition progestins alone, such as norethisterone acetate and dienogest [23], given continuously, improve endometriosis-related pain symptoms. Oral contraceptive pill containing dienogest inhibits ovulation and maintains cyclic menstrual bleeding. Still it is highly effective in controlling pain associated with the disease, in particular dysmenorrhea, chronic pelvic pain, and dyspareunia, and in improving quality of life [24].

GnRH agonist therapy, inducing amenorrhea and anovulation, is a highly effective treatment option for many subjects with endometriosis, but it is accompanied by nonnegligible side effects of bone loss and menopausal symptoms. For these reasons, its use should not exceed six months [25].

### 2.3. Effect of Menstruation Inhibition on Endometrioma Development

In endometriosis, the cyclical bleeding could be associated with a retrograde fall of blood containing cytokines and other inflammation mediators secreted by ischaemic endometrium. Accordingly, avoiding menstrual bleeding may improve disease control and can increase the effect of medical treatments on pain.

There is no data in the literature reporting the effect of tubal sterilization and the subsequent risk of developing an endometrioma, but there are cases describing endometriosis recurrence after hysterectomy. Likely, the recurrence of disease after hysterectomy can be due to persistence of the disease [26].

Following conservative surgery the continuous instead of cyclic administration of a hormonal contraceptive, avoiding cyclic menstrual flows, is associated with a greater reduction of endometrioma recurrence [27, 28] and a more effective pain management [24, 29]. These findings empirically suggest that the development of endometrioma not only is dependent on ovulation, but also is intrinsically connected with menstruation. These findings should be taken into consideration when counseling the patients on the most effective therapies capable of avoiding recurrence of clinical disease, pain, and associated infertility.

## 3. Endometriosis-Associated Ovarian Cancer

### 3.1. Pathogenesis of Endometriosis-Associated Ovarian Cancer (EAOC)

#### 3.1.1. Prevalence of Ovarian Cancer

Ovarian cancer remains the most lethal gynaecological tumor and its prevalence is increasing among gynaecologic malignancies, counting worldwide for 3.7% of all female cancers and for 4.2% of all oncologic deaths in women every year [30]. The reason for this poor prognosis lies mostly in the lack of early detection strategies and effective treatments at the progression after surgical cytoreduction and front-line chemotherapy [31].

More than 90% of ovarian tumors have epithelial origin, while the rest of ovarian malignancies arise from germ cells or granulosa-theca cells. Of all epithelial tumors, about 60–70% are serous, 5% are mucinous, and 15% are either endometrioid and clear-cell [32]. Based on the histopathologic and molecular genetic alterations, serous ovarian carcinomas can be further subdivided in high- (90–95%) and low-grade (5–10%) subgroups, making up a total of 5 main types of epithelial ovarian cancers with essential differences in epidemiological and genetic risk factors, precursor lesions, patterns of spread, oncogenetic mechanisms, and prognosis [33].

#### 3.1.2. Pathogenesis of Epithelial Ovarian Cancer

Notably, each histotype shows a morphological differentiation resembling the normal cells that line the fallopian tube (serous), endocervix (mucinous), endometrium (endometrioid), and vagina (clear-cell).

The epidemiological and genetic risk factors, precursor lesions, pattern of progression, and molecular events during oncogenesis strongly suggest that mucinous cancers are unique among epithelial ovarian malignancies and their development is scarcely influenced by any reproductive factor and thus is not reduced by anovulation and pregnancy [34]. For all these reasons, the possibilities of prevention during reproductive life are scarce.

Like those proposed for endometriomas, there are various hypotheses formulated to explain ovarian carcinogenesis for serous, endometrioid, and clear-cell tumors but none is perfectly in agreement with epidemiological observations.

Fathalla [35] proposed that ovulation implies a sort of inflammatory response, with cellular infiltration as well as the release of cytokines and chemokines. This chronic mechanism of cellular infiltration and cytokines release, along with the exposure of the ovarian surface mesothelium to repeated trauma and repair processes, may induce DNA damage and result in malignant degeneration. The fundamental issue unclarified in this hypothesis is that the reduction in ovarian cancer risk achieved with tubal sterilization is not due to ovulation inhibition [36]. Furthermore, diseases causing chronic anovulation, such as polycystic ovarian syndrome, have no such protective effect [37].

Recent investigations have instead suggested that a substantial number of traditionally considered primary serous, endometrioid, and clear-cell ovarian cancers originate in the fallopian tube and the endometrium and involve the ovary secondarily [33].

**Figure 3:**
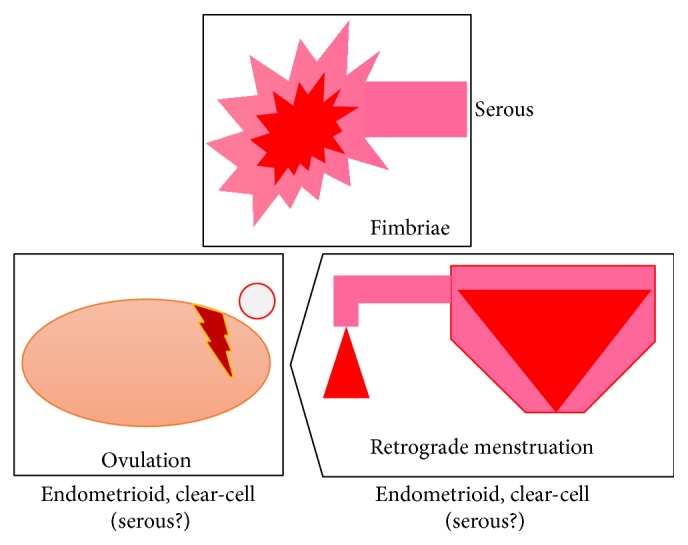
The three “actors” involved in the pathogenesis of serous, endometrioid, and clear-cell ovarian cancers: the retrograde menstruation, the ovulation, and the fimbriae.

Studies of women with BRCA1 or BRCA2 mutations undergoing risk reducing salpingooophorectomy have highlighted the distal fallopian tube as a common (80%) site of tumor origin and additional studies of unselected women with pelvic serous carcinoma have demonstrated that serous tubal intraepithelial carcinoma (STIC) may precede a significant percentage of these tumors [38]. The theory of the pathogenesis from STIC at the fimbriated end of the Fallopian tube is not confirmed by the fact that ovarian serous cancer is prevented by tubal ligation, a procedure during which the ampulla with its fimbriated end is usually preserved [39]. Vercellini et al. [40] recently unified these theories, proposing the “incessant menstruation” theory: the iron-induced oxidative stress derived from retrograde menstruation. The fimbriae, floating in bloody peritoneal fluid, are exposed to the action of catalytic iron and to the genotoxic effect of reactive oxygen species, generated from haemolysis of erythrocytes by pelvic macrophages. This would explain also the distal site of tubal intraepithelial neoplasia. In their opinion the likelihood of developing epithelial ovarian cancer may be influenced not by the lifetime number of ovulations, but by that of menstruations. The three “actors” involved in the pathogenesis of serous, endometrioid, and clear-cell ovarian cancers (the retrograde menstruation, the ovulation, and the fimbriae) are schematically represented in Figure 3.

#### 3.1.3. Endometriosis-Associated Ovarian Cancer (EAOC)

The relationship between endometriosis and ovarian cancer was firstly described by Sampson in 1927 [5]. Sampson proposed the following criteria for diagnosing the carcinomatous development in endometriosis: (1) coexistence of carcinoma and endometriosis within the same ovary, (2) a similar histological pattern, and (3) exclusion of a second malignant tumor elsewhere. In 1953, Scott has added a fourth criterion, which is the demonstration of a histology-proven transition from benign endometriosis to cancer [41]. Since then, a considerable number of studies have indicated an increased risk of epithelial ovarian cancer among women with endometriosis [42–45], with a prevalence of ovarian cancer ranging from 0.7% to 17% of women with endometriosis [4].

Endometriosis was associated with a significantly increased risk of clear-cell and endometrioid invasive ovarian cancer with an odds ratio ranging from 3.7 to 35.4. A recent pooled analysis showed also a 2-fold increased risk for low-grade serous carcinomas [46]. No association was reported between endometriosis and mucinous or high-grade serous ovarian cancer or borderline tumors of either subtype [46]. While endometriosis does share some aspects of malignancy, such as increased growth and vascularization and tissue invasion, in this disease the pivotal characteristics of cancer (monoclonal expansion, genetic abnormalities, and replicative advantage) remain to be defined [47]. Experimental data seems to be consistent with the progression model for carcinogenesis from the benign precursor to ovarian cancer but they could not be unequivocally replicated.

EAOC is described as an ovarian cancer having both cancer cells and endometriosis in the same ovary, presence of cancer in one ovary, and endometriosis in second ovary or presence of ovarian cancer and pelvic endometriosis.

**Figure 4:**
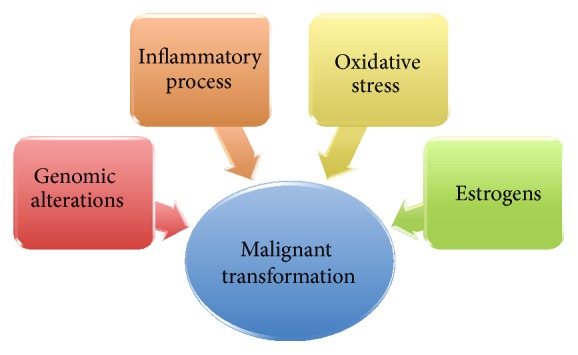
Different mechanisms involved in the malignant transformation of an endometrioma.

Recent molecular studies have linked endometriosis with ovarian cancer through pathways related to oxidative stress, inflammation, and hyperestrogenism and finally to genomic alterations [48] (Figure 4).

On the bases of the gene expression profile and some histopathological and clinical features, ovarian cancer has been divided into two distinct subgroups: type I and type II ovarian cancers. Type I ovarian cancer includes low-grade and borderline serous, endometrioid, mucinous, and clear-cell carcinomas. The most frequent mutations in this type of tumors involve KRAS, BRAF, ERBB2, PTEN, PIK3CA, b-catenin gene (CTNNB1), and ARID1A and PPP2R1A genes. The other tumors, including high-grade serous, mixed malignant mesodermal, carcinosarcomas, and undifferentiated tumors are classified as type II ovarian cancer. Type II tumors, which include the majority of epithelial tumors, are more aggressive and in up to 95% of patients TP53 is affected by a mutation [49, 50]. For other researchers the classification of ovarian cancers into just two types is artificial and limits the progress in understanding the biology of the disease: they have proposed 5 clinically, morphologically, and molecularly different classes of the disease, basing on different molecular abnormalities (high-grade serous: BRCA, p53; low-grade serous: BRAF, KRAS; mucinous: KRAS, HER2; endometrioid: PTEN, ARID1A; and clear-cell: HNF1, ARID1A) [33]. Notably, recent genome sequencing studies reported frequent mutations of ARID1A and PIK3CA genes and moderate mutations of PPP2R1A and KRAS in ovarian clear-cell carcinomas [51, 52] and frequent mutations of PTEN, CTNNB1, and KRAS in endometrioid cancer [53, 54]. In accordance with these results, activation of oncogenic KRAS and PI3K pathways and inactivation of tumor suppressor genes PTEN and ARID1A are suggested pathogenic mechanisms for clear-cell and endometrioid ovarian cancers [55, 56].

Histologically benign endometriosis may harbor genetic abnormalities that predispose for malignant transformation. The malignant transformation progresses gradually from benign endometriosis to carcinoma through intermediary endometriotic lesions, such as atypical endometriosis. Mutations of ARID1A have been demonstrated in atypical endometriosis, indicating the fact that ARID1A mutations are an early event in the pathogenesis of EAOC. On the other hand, no alterations of ARID1A expression were found in the distal nonatypical endometriotic tissue of the same patients [57]. These data suggest that ARID1A is a tumor suppressor gene, whose loss of expression leads to a process of precancerous transition. However, it is widely discussed at which stage of ovarian cancer development ARID1A mutations occur. In fact, several studies indicated that loss of ARID1A expression is also observable in some cases of nonatypical endometriomas [58].

Activation of the PI3K/AKT pathway (through mutation of PIK3CA and AKT or inactivating mutations of PTEN) is a frequent event in clear-cell and endometrioid ovarian cancers. Activating mutations in PIK3CA have been described to occur in 33–40% of clear-cell ovarian cancer, and loss of PTEN expression has been found in 40% of clear-cell ovarian cancers and AKT2 amplification in 14% [59, 60]. At the same time, several studies described PI3K/AKT pathway activation in endometriosis [61–64]; particularly inactivation of PTEN was detected in more than 75% of EAOCs [65] and in about 15% of endometriotic lesions [66]. In the endometriotic lesions, PI3K/AKT pathway regulates FOXO1 protein, a member of the forkhead-box O family, and the decidua-specific gene IGF binding protein-1 (IGFBP-1), which are both involved in the decidualization of endometrial cells. Interestingly, both inhibition of PI3K and AKT led to increasing nuclear FOXO1 and IGFBP1 levels in response to treatment with medroxyprogesterone acetate and dibutyryl cAMP, supporting evidence that the increased PI3K/AKT pathway is involved in the reduced decidual response in endometriosis [67].

**Figure 5:**
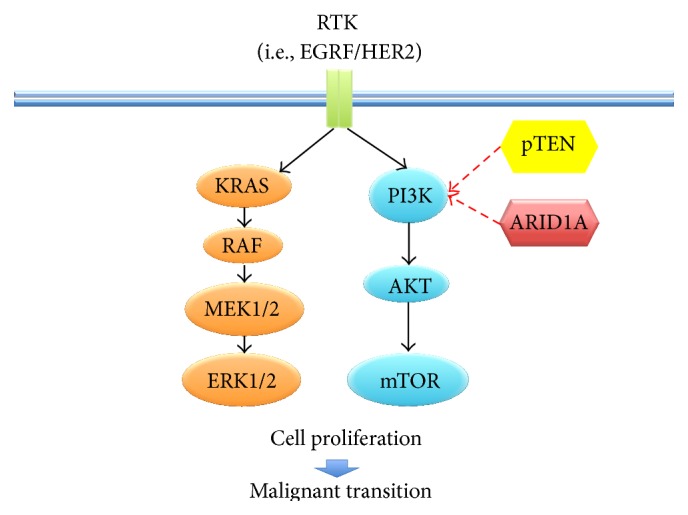
Molecular pathways involved in endometriosis-associated ovarian cancer pathogenesis from endometriotic lesions. In particular, activation of oncogenic KRAS and PI3K pathways and inactivation of tumor suppressor genes PTEN and ARID1A.

Mutations of the gene KRAS were found in 10–20% of EAOCs [68–70]. In a more recent study, KRAS mutations were identified in 29% of endometriosis-associated endometrioid cancers but in only 3% of tumors in which endometriosis was not identified, supporting the hypothesis that KRAS mutations have an important role only in EAOCs [71]. Furthermore, Dinulescu et al. forced expression of oncogenic KRAS or conditional PTEN deletion in ovarian surface epithelium of a mouse model obtaining preneoplastic ovarian lesions with an endometrial glandular morphology, while the combination of both gave rise to invasive and widely metastatic endometrioid ovarian cancer [72]. The pathways involved in EAOC pathogenesis from endometriomas are reassumed in Figure 5.

Chronic inflammation has been demonstrated in the establishment and progression of endometriosis, through the secretion of growth factors and proinflammatory cytokines, including matrix metalloproteinase- (MMP-) 3, interleukin- (IL-) 6, intercellular adhesion molecule-1, tumor necrosis factor- (TNF-) alpha, and IL-8, inducing proliferation of peritoneal macrophages and mesothelial cells. The inflammatory state increases during the menstrual phase, probably as the consequence of the irritative stimulus induced by retrograde menstruation [73].

Numerous studies have demonstrated an association between hyperestrogenism and gynecologic malignancies, including cancers of the breast, endometrium, and ovary [45]. Several mechanisms facilitate the accumulation of an excess of estrogens in endometriomas. The enzyme aromatase is highly present in endometriomas [74]. Aromatase catalyzes the conversion of androstenedione and testosterone, derived from ovarian and adrenal sources, to estrone and estradiol (E2), respectively. Furthermore, in endometriomas the enzyme 17*β*-hydroxysteroid-dehydrogenase (17*β*-HSD) type 2 is lacking. This enzyme can convert E2 to the less potent estrogen estrone [75]. Excess of E2 can result in cellular proliferation through the stimulation of cytokine production, specifically interleukin- (IL-) 8, and RANTES and also of prostaglandin E2 (PGE2) that can stimulate the activity of aromatase, resulting in a positive feedback loop in favor of hyperestrogenism. This highly proliferative microenvironment in endometrioma presents an enhanced level of reparative activity, with a higher chance for DNA damage and mutations.

Among EAOC, there is an important difference in steroid receptors: the clear-cell subtype typically does not express estrogen (ER) and progesterone receptors (PR), while the endometrioid subtype expresses both of them [76]. Expression of ER could be a pivotal point in the carcinogenic pathway, separating the development of estrogen-dependent carcinomas (endometrioid) from estrogen-independent carcinomas (clear-cell) [77].

According to some authors, patients with EAOC have a lower stage of cancer, a distribution of histological subtypes that differs from the general population, predominantly lower-grade endometriosis lesions, and significantly better overall survival as compared with other ovarian carcinomas [48]. The possible explanation for this evidence is that benign symptomatic disease leads to an increased number of examinations and scans, which in turn may lead to an earlier diagnosis of OC. Conversely, in other trials endometriosis per se does not appear to predict prognosis especially in clear-cell and endometrioid tumors, not resulting in a prolonged overall survival [78].

### 3.2. Effect of Ovulation Inhibition on Ovarian Cancer Development

Ovarian cancer risk is higher in nulliparous women, with a history of “incessant ovulation” [79]. Long-term artificial inhibition of ovulation can be obtained during reproductive life with the use of hormonal contraceptives. About one-third of ovarian cancers are prevented by ever use of a hormonal contraceptive [36, 80]. The longer the woman uses hormonal contraceptives the greater is the reduction in ovarian cancer risk, with the use for about 15 years reducing the risk of ovarian cancer of about 50%. Interestingly, this protective effect continues for more than 30 years after hormonal contraceptive discontinuation, with a progressive reduction over time. Also the mortality from ovarian cancer is reduced in hormonal contraceptives users with a RR of 0.4 (95% CI, 0.3–0.6), which progressively declines in relation to total duration of use [81]. The mechanism of this protection is not fully understood but it seems to be directly connected to the lifetime number of ovulatory cycles inhibited [82]. However, it seems that hormonal contraceptives provide a stronger protective effect than expected from anovulatory action alone.

The preventive effect of hormonal contraceptives is different among several histological subtypes: a lower degree of risk reduction being observed for mucinous invasive cancers. After 5 years of hormonal contraceptive use, Beral et al. [82] showed a risk reduction of 22.1% for serous, of 27.1% for endometrioid, and of 21.3% for clear-cell carcinomas, but only a nonsignificant 6.7% reduction for mucinous ones. A study has estimated that women with endometriosis benefit most from long-term inhibition of ovulation with hormonal contraceptives, experiencing a risk reduction of about 80% following 10 years of use [83].

### 3.3. Effect of Menstruation Inhibition on Ovarian Cancer Development

An in vivo model of absence of retrograde menstruation is tubal sterilization. A systematic meta-analysis [39] observed a 34% reduction in the risk of ovarian cancer after tubal sterilization (RR 0.66; 95% CI 0.60–0.73). The overall protective effect afforded by tubal ligation is substantially similar to that observed with hormonal contraceptive use. Also regarding the histotypes, the effect appears to be the same as for hormonal contraceptive use, in particular, the greatest for endometrioid cancers (RR 0.40; 95% CI 0.30–0.53), slightly reduced but significant for serous subtypes (RR 0.73; 95% CI 0.63–0.85), and absent for the mucinous subgroup (RR 0.92; 95% CI 0.66–1.30).

Consistently, also hysterectomy decreased the risk of ovarian cancer (RR 0.64, 95% CI 0.48–0.85) [45, 84].

Unfortunately, there are no data on ovarian cancer risk with the use of hormonal contraceptives given in a continuous fashion, in order to avoid cyclic menstrual bleedings. Theoretically such hormonal contraceptive use should be associated with a further reduction of ovarian cancer risk.

## 4. Conclusions

Endometriosis is a relatively common disease in the general population, affecting about one in ten women. Women with endometriosis are at doubled-tripled risk of specific gonadal malignancies. The ovarian cancer subtypes most frequently associated with endometriosis are clear-cell and endometrioid carcinomas that represent about 30% of total ovarian cancers. Recent data demonstrated a doubled risk also for low-grade serous ovarian cancers (about 5% of ovarian cancers) while mucinous (about 5%) and high-grade serous (about 60%) cancers appear not to be associated with the disease. This last subtype is the most common epithelial ovarian cancer, often presenting with an advanced disease stage at diagnosis for the very early transcoelomic pattern of spread, resulting in the poorest prognosis [33].

In our review, we show how both endometriomas and EAOCs are intrinsically and similarly dependent by the number of ovulations and retrograde menstruations during a woman reproductive life. Reduced number of pregnancies and late pregnancies and reduced time of lactation have led to a greater exposure of women to risk factors for endometriosis and EAOC, such as ovulation and cyclic menstrual bleeding, prompting an increased prevalence of these diseases.

Multiple pregnancies reduce the risk for both the disorders. Tubal sterilization, which avoids menstrual reflux in peritoneal cavity, is associated with a similar decrease of ovarian cancer risk (EAOCs and high-grade serous cancers) and similarly hysterectomy reduces the risk of ovarian cancer. Furthermore, the risk for EAOCs and high-grade serous cancers is also dramatically reduced by the use of hormonal contraceptives that reduce the exposure to both ovulation and cyclic menstrual reflux during reproductive life. Similarly the continuous use of hormonal contraceptives particularly in a continuous fashion reduces the risk of recurrence after the conservative surgery of an ovarian endometrioma.

For all these reasons, in patients with ovarian endometriosis, methods to inhibit ovulation and/or to reduce retrograde menstruation should be strongly encouraged if indicated, for the possibility to protect from endometrioma recurrence, from the increased risk of transformation in EOACs, and from the general incidence of high-grade serous ovarian cancers, although probably similar to that of the general population.

The pathogenetic transformation from endometriosis to ovarian cancer is not fully understood, but it seems mainly related to the cooperation of oxidative stress, inflammation, hyperestrogenism, and specific genomic alterations. A pathogenetic view starting from endometrioma and atypical endometrioma to EAOC seems plausible and supported by the same gene mutations observed in atypical endometriosis and EAOC.

Atypical endometrioma with atypia and/or hyperplasia is a relatively common figure in the histological specimens of our surgeries (about one in twelve). Likely an increased awareness to the characteristics of this entity will improve the early detection of patients who are at highest risk of EOAC and will provide further insights into this issue.

There are some limitations about the data reviewed in this paper. Firstly, the present study is a narrative review that tends to be mainly descriptive, does not involve a systematic search of the literature, and often focuses on a subset of studies in an area chosen based on availability or author selection, consisting in a possible selection bias. They can also be confusing at times, particularly if similar studies have diverging results and conclusions. Furthermore, this review is not based on prospective randomized controlled trials but mainly on large observational population studies. For these reason, the results of the present review should be interpreted with caution.

To conclude, the carcinogenetic potential of endometrioma is a continuous source of interest and its study may help to clarify the pathogenesis of ovarian cancer and to better adopt effective preventive strategies.

## References

[1] Bulun S. E. (2009). Endometriosis. *The New England Journal of Medicine*.

[2] Lapp T. (2000). ACOG issues recommendations for the management of endometriosis. *American Family Physician*.

[3] Grandi G., Xholli A., Ferrari S., Cannoletta M., Volpe A., Cagnacci A. (2013). Intermenstrual pelvic pain, quality of life and mood. *Gynecologic and Obstetric Investigation*.

[4] Heidemann L. N., Hartwell D., Heidemann C. H., Jochumsen K. M. (2014). The relation between endometriosis and ovarian cancer—a review. *Acta Obstetricia et Gynecologica Scandinavica*.

[5] Sampson J. A. (1927). Peritoneal endometriosis due to menstrual dissemination of endometrial tissue into the peritoneal cavity. *American Journal of Obstetrics & Gynecology*.

[6] Sanfilippo J. S., Wakim N. G., Schikler K. N., Yussman M. A. (1986). Endometriosis in association with uterine anomaly. *American Journal of Obstetrics & Gynecology*.

[7] Salamanca A., Beltran E. (1995). Subendometrial contractility in menstrual phase visualized by transvaginal sonography in patients with endometriosis. *Fertility and Sterility*.

[8] Jenkins S., Olive D. L., Haney A. F. (1986). Endometriosis: pathogenetic implications of the anatomic distribution. *Obstetrics and Gynecology*.

[9] Cho M. K., Kim C. H., Oh S. T. (2009). Endometriosis in a patient with Rokitansky-Kuster-Hauser syndrome. *Journal of Obstetrics and Gynaecology Research*.

[10] Nisolle M., Donnez J. (1997). Peritoneal endometriosis, ovarian endometriosis, and adenomyotic nodules of the rectovaginal septum are three different entities. *Fertility and Sterility*.

[11] Kurman R. J., Shih I.-M. (2010). The origin and pathogenesis of epithelial ovarian cancer: a proposed unifying theory. *The American Journal of Surgical Pathology*.

[12] Hughesdon P. E. (1957). The structure of endometrial cysts of the ovary. *Journal of Obstetrics and Gynecology British Empire*.

[13] Brosens I. A., Puttemans P. J., Deprest J. (1994). The endoscopic localization of endometrial implants in the ovarian chocolate cyst. *Fertility and Sterility*.

[14] Nezhat F., Nezhat C., Allan C. J., Metzger D. A., Sears D. L. (1992). Clinical and histologic classification of endometriomas. Implications for a mechanism of pathogenesis. *Journal of Reproductive Medicine*.

[15] Vercellini P., Somigliana E., Vigano P., Abbiati A., Barbara G., Fedele L. (2009). ‘Blood on the Tracks’ from corpora lutea to endometriomas. *BJOG*.

[16] Nisolle M., Donnez J. (1997). Peritoneal endometriosis, ovarian endometriosis, and adenomyotic nodules of the rectovaginal septum are three different entities. *Fertility and Sterility*.

[17] Tanase Y., Furukawa N., Kobayashi H., Matsumoto T. (2013). Malignant transformation from endometriosis to atypical endometriosis and finally to endometrioid adenocarcinoma within 10 years. *Case Reports in Oncology*.

[18] Czernobilsky B., Morris W. J. (1979). A histologic study of ovarian endometriosis with emphasis on hyperplastic and atypical changes. *Obstetrics and Gynecology*.

[19] Thomas E. J., Campbell I. G. (2000). Evidence that endometriosis behaves in a malignant manner. *Gynecologic and Obstetric Investigation*.

[20] Van Gorp T., Amant F., Neven P., Vergote I., Moerman P. (2004). Endometriosis and the development of malignant tumours of the pelvis: a review of literature. *Best Practice and Research: Clinical Obstetrics and Gynaecology*.

[21] Somigliana E., Vercellini P., Vigano P., Abbiati A., Benaglia L., Fedele L. (2011). Endometriosis and estroprogestins: the chicken or the egg causality dilemma. *Fertility and Sterility*.

[22] Vercellini P., De Matteis S., Somigliana E., Buggio L., Frattaruolo M. P., Fedele L. (2013). Long-term adjuvant therapy for the prevention of postoperative endometrioma recurrence: a systematic review and meta-analysis. *Acta Obstetricia et Gynecologica Scandinavica*.

[23] Köhler G., Faustmann T. A., Gerlinger C., Seitz C., Mueck A. O. (2010). A dose-ranging study to determine the efficacy and safety of 1, 2, and 4 mg of dienogest daily for endometriosis. *International Journal of Gynecology and Obstetrics*.

[24] Grandi G., Xholli A., Napolitano A., Palma F., Cagnacci A. (2015). Pelvic pain and quality of life of women with endometriosis during quadriphasic estradiol valerate/dienogest oral contraceptive a patient-preference prospective 24-week pilot study. *Reproductive Sciences*.

[25] DiVasta A. D., Laufer M. R. (2013). The use of gonadotropin releasing hormone analogues in adolescent and young patients with endometriosis. *Current Opinion in Obstetrics & Gynecology*.

[26] Rizk B., Fischer A. S., Lotfy H. A. (2014). Recurrence of endometriosis after hysterectomy. *Facts, Views & Vision in ObGyn*.

[27] Vlahos N., Vlachos A., Triantafyllidou O., Vitoratos N., Creatsas G. (2013). Continuous versus cyclic use of oral contraceptives after surgery for symptomatic endometriosis: a prospective cohort study. *Fertility and Sterility*.

[28] Seracchioli R., Mabrouk M., Frascà C. (2010). Long-term cyclic and continuous oral contraceptive therapy and endometrioma recurrence: a randomized controlled trial. *Fertility and Sterility*.

[29] Seracchioli R., Mabrouk M., Frascà C., Manuzzi L., Savelli L., Venturoli S. (2010). Long-term oral contraceptive pills and postoperative pain management after laparoscopic excision of ovarian endometrioma: a randomized controlled trial. *Fertility and Sterility*.

[30] Ferlay J., Shin H.-R., Bray F., Forman D., Mathers C., Parkin D. M. (2010). Estimates of worldwide burden of cancer in 2008: GLOBOCAN 2008. *International Journal of Cancer*.

[31] Thigpen T. (2012). A rational approach to the management of recurrent or persistent ovarian carcinoma. *Clinical Obstetrics and Gynecology*.

[32] Mok S. C., Kwong J., Welch W. R. (2007). Etiology and pathogenesis of epithelial ovarian cancer. *Disease Markers*.

[33] Prat J. (2012). New insights into ovarian cancer pathology. *Annals of Oncology*.

[34] Cramer D. W., Xu H. (1995). Epidemiologic evidence for uterine growth factors in the pathogenesis of ovarian cancer. *Annals of Epidemiology*.

[35] Fathalla M. F. (1971). Incessant ovulation—a factor in ovarian neoplasia?. *The Lancet*.

[36] Cibula D., Gompel A., Mueck A. O. (2010). Hormonal contraception and risk of cancer. *Human Reproduction Update*.

[37] Schildkraut J. M., Schwingl P. J., Bastos E., Evanoff A., Hughes C. (1996). Epithelial ovarian cancer risk among women with polycystic ovary syndrome. *Obstetrics and Gynecology*.

[38] Folkins A. K., Jarboe E. A., Roh M. H., Crum C. P. (2009). Precursors to pelvic serous carcinoma and their clinical implications. *Gynecologic Oncology*.

[39] Cibula D., Widschwendter M., Májek O., Dusek L. (2011). Tubal ligation and the risk of ovarian cancer: review and meta-analysis. *Human Reproduction Update*.

[40] Vercellini P., Crosignani P., Somigliana E. (2011). The ‘incessant menstruation’ hypothesis: a mechanistic ovarian cancer model with implications for prevention. *Human Reproduction*.

[41] Scott R. B. (1953). Malignant changes in endometriosis.. *Obstetrics and Gynecology*.

[42] Brinton L. A., Gridley G., Persson I., Baron J., Bergqvist A. (1997). Cancer risk after a hospital discharge diagnosis of endometriosis. *American Journal of Obstetrics & Gynecology*.

[43] Erzen M., Kovacic J. (1998). Relationship between endometriosis and ovarian cancer. *European Journal of Gynaecological Oncology*.

[44] Ogawa S., Kaku T., Amada S. (2000). Ovarian endometriosis associated with ovarian carcinoma: a clinicopathological and immunohistochemical study. *Gynecologic Oncology*.

[45] Melin A., Sparén P., Persson I., Bergqvist A. (2006). Endometriosis and the risk of cancer with special emphasis on ovarian cancer. *Human Reproduction*.

[46] Pearce C. L., Templeman C., Rossing M. A. (2012). Association between endometriosis and risk of histological subtypes of ovarian cancer: a pooled analysis of case-control studies. *The Lancet Oncology*.

[47] Viganó P., Somigliana E., Chiodo I., Abbiati A., Vercellini P. (2006). Molecular mechanisms and biological plausibility underlying the malignant transformation of endometriosis: a critical analysis. *Human Reproduction Update*.

[48] Worley M. J., Welch W. R., Berkowitz R. S., Ng S.-W. (2013). Endometriosis-associated ovarian cancer: a review of pathogenesis. *International Journal of Molecular Sciences*.

[49] Kurman R. J. (2013). Origin and molecular pathogenesis of ovarian high-grade serous carcinoma. *Annals of Oncology*.

[50] Kurman R. J., Shih I.-M. (2008). Pathogenesis of ovarian cancer: lessons from morphology and molecular biology and their clinical implications. *International Journal of Gynecological Pathology*.

[51] Wiegand K. C., Shah S. P., Al-Agha O. M. (2010). ARID1A mutations in endometriosis-associated ovarian carcinomas. *The New England Journal of Medicine*.

[52] Jones S., Wang T.-L., Shih I.-M. (2010). Frequent mutations of chromatin remodeling gene ARID1A in ovarian clear cell carcinoma. *Science*.

[53] Kolasa I. K., Rembiszewska A., Janiec-Jankowska A. (2006). PTEN mutation, expression and LOH at its locus in ovarian carcinomas. Relation to TP53, K-RAS and BRCA1 mutations. *Gynecologic Oncology*.

[54] Palacios J., Gamallo C. (1998). Mutations in the beta-catenin gene (CTNNB1) in endometrioid ovarian carcinomas. *Cancer Research*.

[55] Madore J., Ren F., Filali-Mouhim A. (2010). Characterization of the molecular differences between ovarian endometrioid carcinoma and ovarian serous carcinoma. *The Journal of Pathology*.

[56] Banz C., Ungethuem U., Kuban R.-J., Diedrich K., Lengyel E., Hornung D. (2010). The molecular signature of endometriosis-associated endometrioid ovarian cancer differs significantly from endometriosis-independent endometrioid ovarian cancer. *Fertility and Sterility*.

[57] Cornen S., Adelaide J., Bertucci F. (2012). Mutations and deletions of ARID1A in breast tumors. *Oncogene*.

[58] Yamamoto S., Tsuda H., Takano M., Tamai S., Matsubara O. (2012). Loss of ARID1A protein expression occurs as an early event in ovarian clear-cell carcinoma development and frequently coexists with PIK3CA mutations. *Modern Pathology*.

[59] Popovic R., Licht J. D. (2012). Emerging epigenetic targets and therapies in cancer medicine. *Cancer Discovery*.

[60] Samartzis E. P., Noske A., Dedes K. J., Fink D., Imesch P. (2013). ARID1A mutations and PI3K/AKT pathway alterations in endometriosis and endometriosis-associated ovarian carcinomas. *International Journal of Molecular Sciences*.

[61] Liu P., Cheng H., Roberts T. M., Zhao J. J. (2009). Targeting the phosphoinositide 3-kinase pathway in cancer. *Nature Reviews Drug Discovery*.

[62] Yin X., Pavone M. E., Lu Z., Wei J., Kim J. J. (2012). Increased activation of the PI3K/AKT pathway compromises decidualization of stromal cells from endometriosis. *The Journal of Clinical Endocrinology & Metabolism*.

[63] Laudanski P., Szamatowicz J., Kowalczuk O., Kuźmicki M., Grabowicz M., Chyczewski L. (2009). Expression of selected tumor suppressor and oncogenes in endometrium of women with endometriosis. *Human Reproduction*.

[64] Sato N., Tsunoda H., Nishida M. (2000). Loss of heterozygosity on 10q23.3 and mutation of the tumor suppressor gene PTEN in benign endometrial cyst of the ovary: possible sequence progression from benign endometrial cyst to endometrioid carcinoma and clear cell carcinoma of the ovary. *Cancer Research*.

[65] Orezzoli J. P., Russell A. H., Oliva E., Del Carmen M. G., Eichhorn J., Fuller A. F. (2008). Prognostic implication of endometriosis in clear cell carcinoma of the ovary. *Gynecologic Oncology*.

[66] Gounaris I., Charnock-Jones D. S., Brenton J. D. (2011). Ovarian clear cell carcinoma-bad endometriosis or bad endometrium?. *Journal of Pathology*.

[67] Engelman J. A. (2009). Targeting PI3K signalling in cancer: opportunities, challenges and limitations. *Nature Reviews Cancer*.

[68] Auner V., Kriegshäuser G., Tong D. (2009). KRAS mutation analysis in ovarian samples using a high sensitivity biochip assay. *BMC Cancer*.

[69] Mayr D., Hirschmann A., Löhrs U., Diebold J. (2006). KRAS and BRAF mutations in ovarian tumors: a comprehensive study of invasive carcinomas, borderline tumors and extraovarian implants. *Gynecologic Oncology*.

[70] Geyer J. T., López-García M. A., Sánchez-Estevez C. (2009). Pathogenetic pathways in ovarian endometrioid adenocarcinoma: a molecular study of 29 cases. *The American Journal of Surgical Pathology*.

[71] Stewart C. J. R., Leung Y., Walsh M. D., Walters R. J., Young J. P., Buchanan D. D. (2012). KRAS mutations in ovarian low-grade endometrioid adenocarcinoma: association with concurrent endometriosis. *Human Pathology*.

[72] Dinulescu D. M., Ince T. A., Quade B. J., Shafer S. A., Crowley D., Jacks T. (2005). Role of K-ras and Pten in the development of mouse models of endometriosis and endometrioid ovarian cancer. *Nature Medicine*.

[73] Kyama C. M., Overbergh L., Debrock S. (2006). Increased peritoneal and endometrial gene expression of biologically relevant cytokines and growth factors during the menstrual phase in women with endometriosis. *Fertility and Sterility*.

[74] Zeitoun K. M., Bulun S. E. (1999). Aromatase: a key molecule in the pathophysiology of endometriosis and a therapeutic target. *Fertility and Sterility*.

[75] Zeitoun K., Takayama K., Sasano H. (1998). Deficient 17*β*-hydroxysteroid dehydrogenase type 2 expression in endometriosis: failure to metabolize 17*β*-estradiol. *Journal of Clinical Endocrinology and Metabolism*.

[76] Soslow R. A. (2008). Histologic subtypes of ovarian carcinoma: an overview. *International Journal of Gynecological Pathology*.

[77] Mandai M., Yamaguchi K., Matsumura N., Baba T., Konishi I. (2009). Ovarian cancer in endometriosis: molecular biology, pathology, and clinical management. *International Journal of Clinical Oncology*.

[78] Cuff J., Longacre T. A. (2012). Endometriosis does not confer improved prognosis in ovarian carcinoma of uniform cell type. *The American Journal of Surgical Pathology*.

[79] Högnäs E., Kauppila A., Pukkala E., Tapanainen J. S. (2014). Cancer risk in women with 10 or more deliveries. *Obstetrics and Gynecology*.

[80] Hannaford P. C., Selvaraj S., Elliott A. M., Angus V., Iversen L., Lee A. J. (2007). Cancer risk among users of oral contraceptives: cohort data from the Royal College of General Practitioners' oral contraception study. *British Medical Journal*.

[81] Vessey M., Yeates D., Flynn S. (2010). Factors affecting mortality in a large cohort study with special reference to oral contraceptive use. *Contraception*.

[82] Collaborative Group on Epidemiological Studies of Ovarian Cancer (2008). Ovarian cancer and oral contraceptives: collaborative reanalysis of data from 45 epidemiological studies including 23,257 women with ovarian cancer and 87,303 controls. *The Lancet*.

[83] Modugno F., Ness R. B., Allen G. O., Schildkraut J. M., Davis F. G., Goodman M. T. (2004). Oral contraceptive use, reproductive history, and risk of epithelial ovarian cancer in women with and without endometriosis. *American Journal of Obstetrics & Gynecology*.

[84] Green A., Purdie D., Green L., Dick M. L., Bain C., Siskind V. (1997). Tubal sterilisation, hysterectomy and decreased risk of ovarian cancer. Survey of Women's Health Study Group. *Australian and New Zealand Journal of Public Health*.

